# Reduced tumorigenicity and drug resistance through the downregulation of octamer-binding protein 4 and Nanog transcriptional factor expression in human breast stem cells

**DOI:** 10.3892/mmr.2014.2972

**Published:** 2014-11-18

**Authors:** ZHENG-JIE HUANG, JUN YOU, WEI-YUAN LUO, BAI-SHENG CHEN, QING-ZHAO FENG, BING-LIN WU, LONG JIANG, QI LUO

**Affiliations:** 1 Department of Surgical Oncology, The First Affiliated Hospital of Xiamen University, Xiamen, Fujian 361003, P.R. China; 2 Department of Surgical Oncology, First Clinical Medical College of Fujian Medical University, Fuzhou, Fujian 350004, P.R. China

**Keywords:** breast cancer stem cells, isolation and identification, octamer-binding protein 4, Nanog, tumorigenicity, drug resistance

## Abstract

Breast cancer is the most common type of malignancy among females. Previous studies examining breast cancer tissue have demonstrated the presence of stem cells, and have detected octamer-binding protein 4 (Oct4) and Nanog transcription factor expression. In the present study, breast cancer stem cells (CSCs) were isolated and enriched from MDA-MB-231 breast cancer cell lines, and were defined as MDA-MB-231 stem cells using flow cytometry. The expression of Oct4 and Nanog in breast CSCs were detected by quantitative polymerase chain reaction and western blotting. RNA interference (RNAi) was used in order to downregulate the expression of Oct4 and Nanog. Drug resistance and tumor-initiating capability following *in vivo* injection of MDA-MB-231 stem cells transduced with negative RNAi, Oct4 RNAi and Nanog RNAi were compared with that of MDA-MB-231 stem cells without siRNA transfection as a control group. In addition the capability of MDA-MB-231 breast cancer cells to initiate tumor formation in mice was compared with that of MDA-MB-231 stem cells. A paclitaxel inhibition test was also conducted in order to detect resistance of MDA-MB-231 breast cancer stem cells to this treatment. The MDA-MB-231 stem cells were revealed to exhibit elevated percentages of the cluster of differentiation (CD)44^+^CD24^−/low^ subset, high tumorigenicity and resistance to chemotherapy, all of which are characteristic stem cell properties. In addition, the MDA-MB-231 stem cells were more tumorigenic *in vivo*. Furthermore, the breast CSCs also expressed high levels of the Oct4 and Nanog transcription factors. Therefore, downregulation of Oct4 or Nanog expression may reduce chemotherapeutic drug resistance and tumorigenicity in breast CSCs. In conclusion, Oct4 and Nanog expression may be a key factor in the development of resistance to chemotherapy and tumor growth of breast CSCs. This finding indicates that Oct4 or Nanog-targeted therapy may be a promising means of overcoming resistance to chemotherapy and inhibiting tumor growth in breast cancer treatment.

## Introduction

In recent years, there has been an increasing focus on the cancer stem cell (CSC) hypothesis. This hypothesis identifies a small subset of cancer cells that constitute the bulk of self-sustaining cells with a unique capacity for self-renewal, which cause the development of heterogeneous lineages during tumor formation (1–5). Assays that examine CSC activity require evaluation of the self-renewal and tumor propagation abilities of the cells (3). An increasing number of CSCs in solid tumors have been recognized through sorting cancer cells in serum-free suspension culture conditions and identifying the CSCs on the basis of differential expression of surface markers combined with *in vivo* propagation of tumorogenecity (6–9).

Breast cancer, the most common type of malignancy among females, has an increasing incidence, with an annual growth rate of 3% in China, and is the primary cause of cancer-associated mortality among urban females (10). Tumorigenic breast cancer cells with stem cell properties have been isolated and identified in breast carcinoma lesions (11,12). Due to the limited number of cells within the breast tumor reservoir and the location of the cells within the tumor interstitium, breast CSCs are able to develop resistance to drugs and evade chemotherapy, resulting in disease relapse, even if the primary lesion has been eradicated (13,14). Therefore, investigation of novel drug resistance mechanisms that target stem cells is important to improve the current therapeutic strategies for treating breast cancer.

Octamer-binding protein 4 (Oct4) and Nanog, two of the transcriptional factors that exert key roles in the maintenance of self-renewal and pluripotency in human embryonic stem cells, have been recently observed to be expressed in numerous types of cancer cell line and tissue, and have been associated with aggressive tumors (15–19). Furthermore, downregulation of Oct4 and Nanog has been shown to promote stem cell differentiation and inhibit tumor development (20–22). A number of studies have revealed that Oct4 and Nanog are detected at high levels in human breast cancer tissues, which indicates the critical roles of Oct4 and Nanog in breast stem cell state maintenance and escape from conventional chemotherapy (23,24). However, the underlying molecular mechanism by which Oct4 and Nanog mediate the drug resistance response to chemotherapy in breast CSCs remains to be elucidated.

In the present study, breast CSCs were isolated from MDA-MB-231 breast cancer cells using a serum-free suspension culture, which characterizes the differential expression of cluster of differentiation 44 (CD44) and CD24 on the CSC cell surface combined with the capacity of CSCs to generate novel tumors when injected into a congenetic animal model. Subsequently, the differential expression of Oct4 and Nanog mRNA in the isolated mammosphere MDA-MB-231 breast CSCs (defined as MDA-MB-231 stem cells) and the MDA-MB-231 breast cancer cells was examined. The critical relevance of Oct4 and Nanog with breast CSC therapeutic response to chemotherapy was also investigated.

## Materials and methods

### Ethics

This study was approved by the Institutional Ethics Committee of the First Affiliated Hospital of Xiamen University (Xiamen, China) and was in compliance with national legislation and the Declaration of Helsinki guidelines. All animal experiments were approved by the Animal Care and Use Committee of Xiamen University. Animal care was in accordance with the Regulations for the Administration of Affairs Concerning Experimental Animals of Xiamen University.

### Cell lines and in vitro propagation of human breast stem cells in serum-free culture

MDA-MB-231 human breast cancer cell lines were provided by the Cancer Center of Xiamen Medical College (Xiamen, China). The cells were cultured in differentiation conditions in Dulbecco’s modified Eagle’s medium (DMEM) with 10% fetal bovine serum (FBS). After three days, when the cells covered 90% of the plate, adherent cells were dissociated by incubation in 0.25% trypsin-ethylenediaminetetraacetic acid solution for 1 min at 37°C. MDA-MB-231 cells in the logarithmic growth phase were plated at 10^6^, 10^5^, 10^4^ and 10^3^ cells/ml in serum-free DMEM/F12 (1:1) medium containing 2% B27 (Gibco-BRL, Carlsbad, CA, USA), 20 ng/ml epidermal growth factor (EGF; Sigma-Aldrich, St. Louis, MO, USA) and 20 ng/ml basic fibroblast growth factor (bFGF; Sigma-Aldrich). The cells were cultured in these serum-free conditions as non-adherent mammosphere clusters. Differentiation was induced by culturing mammosphere cells for 12 h in DMEM supplemented with 10% FBS.

### Flow cytometry

The cells were washed twice with phosphate-buffered saline (PBS) and then resuspended in the wash buffer (10^6^ cells/ml). Antibodies against CD44 (fluorescein isothiocyanate-conjugated) and CD24 (phycoerythrin-conjugated) obtained from eBioscience (San Diego, CA, USA), and the corresponding isotype controls were added to the cell suspension, and the cells were incubated at 4°C in the dark for 40 min. Subsequently, the cells were washed twice in 4 ml PBS buffer and then resuspended in 400 μl PBS buffer for flow cytometric analysis. The stained cells were processed using flow cytometry (BD FACSAria™ II; BD Biosciences, Franklin Lakes, NJ, USA). The results were analyzed using FlowJo v.7.6.5 software (TreeStar, Inc., Ashland, OR, USA).

### Paclitaxel inhibition

Cells in a single cell suspension state were seeded in a 96-well plate at a density of 3×10^3^ cells/ml in serum-free DMEM. Subsequently, paclitaxel (Jiangsu Aosaikang Pharmaceutical Co. Ltd., Nanjing, China) was added to the suspension to bring the volume in each plate to 200 μl, and the paclitaxel concentrations to 0.5, 1.0, 2.0, 4.0, 8.0, 16.0 and 32.0 μg/ml, respectively. The zero-adjusting well and control group were then set-up, the former without cells and the latter without paclitaxel. A volume of 20 μl CellTiter-Blue^®^ reagent (Promega Corporation, Madison, WI, USA) was added to every well after 48 h incubation. After 4 h culture at 37°C, the optical density (OD) of each well was then measured by a fluorescence microplate reader (Beckman Institute, Urbana, IL, USA) at 570 nm. The cell inhibition rate was defined as follows: Drug uptake percentage = (OD of control group − OD of experimental group)/(OD of control group)×100%. The data were obtained from three independent experiments each performed in triplicate, in which the median inhibitory concentration (IC_50_) was calculated using ProHits analysis (http://prohitsms.com/Prohits_download/list.php).

### Quantitative (q)PCR

Total RNA extraction from the MDA-MB-231 cells and the MDA-MB-231 stem cells was conducted according to the TRIzol^®^ total RNA extraction kit manufacturer’s instructions. Reverse transcription was performed from 1,000 ng total RNA using the RevertAid First Strand cDNA Synthesis kit according to the manufacturer’s instructions. The gene expression levels relative to those of GAPDH were assessed using qPCR with the ABI-7500 sequence detection system (Applied Biosystems, Inc., Carlsbad, CA, USA) and SYBR-Green chemistry (Shanghai Yingjun Biotechnology Limited Company, Shanghai, China), as follows: Initial denaturation at 95°C for 10 min, followed by 40 cycles of denaturation at 95°C for 15 sec, and annealing and extension at 60°C for 1 min. The human GAPDH, Oct4 and Nanog primer sequences (Sangon Biotech, Shanghai, China) employed are shown in Table I. The reactions were run in triplicate and the generated products were analyzed with the sodium dodecyl sulfate (SDS) software (Bio-Rad Laboratories, Inc., Hercules, CA, USA). The data were evaluated as 2^−ΔΔCt^ values (Ct indicates the cycle threshold). The results are expressed as the normalization ratio of the relative quantities of the Oct4 and Nanog mRNAs to those of the control, and the fold difference to the control was used for the comparison.

### Western blot analysis

The MDA-MB-231 cells and the MDA-MB-231 stem cells were collected and then lysed with radioimmunoprecipitation assay buffer and the protein concentrations within the cells were measured according to the RIPA lysate manufacturer’s instructions (Applygen Technologies Inc., Beijing, China). Equivalent quantities of protein for each sample were separated by SDS-PAGE, transferred to PVDF membranes (Millipore, Billerica, MA, USA) and probed with the following primary antibodies: Rabbit polyclonal antibody against human OCT4 (1:8,000 dilution; cat no.: ab18976), mouse monoclonal antibody against human Nanog (1:1,000 dilution; cat no.: ab89500) and mouse monoclonal antibody against human GAPDH (1:3,000 dilution; cat no.: ab57062). All antibodies were obtained from Abcam (Cambridge, MA, USA). The PVDF membranes were incubated overnight at 4°C with the primary antibodies and then washed three times. A secondary horseradish peroxidase-labeled goat polyclonal antibody against rabbit (1:3,000 dilution; cat no.: ab97200) or a goat polyclonal antibody against mouse (1:4,000 dilution; cat no.: ab97265) were added and incubated for 2 h at room temperature. Immunodetection was performed using an electrochemiluminescent substrate (Pierce Biotechnology, Inc., Rockford, IL, USA) and the Gel Doc XR type imaging system. The intensity of bands was quantified using Image J software (Bio-Rad Laboratories). Three independent experiments, each in triplicate, were conducted in 24-well plates.

### Silencing through RNA interference

To inhibit Oct4 or Nanog expression in the MDA-MB-231 stem cells, RNA interference silencing was performed using RNAfectin Transfection Reagent (Tiangen, Beijing, China) according to the manufacturer’s instructions. All double-stranded siRNAs were designed and synthesized by Qiagen (Valencia, CA, USA). The siRNA sequences are shown in Table II.

The breast CSCs were initially plated in 24-well plates at a density of 2×10^5^ cells/well in DMEM medium, and after 24 h were transfected with 7.5 ng/μl siRNA against Oct4, Nanog or non-targeting siRNA. Cells that had not been transfected served as controls. The cells were harvested 48 h after transfection to calculate the mRNA and protein expression levels.

### In vivo injection of MDA-MB-231 stem cells

A total of 60 four-week-old female NOD/SCID mice with a mean body weight of 25±5 g were purchased from the Experimental Animal Center of Xiamen University (Xiamen, China). All mice were maintained in specific pathogen-free rooms at a certain temperature and humidity, and were provided free access to fresh water and food. A total of 40 of the NOD/SCID mice were randomly divided into eight groups by drawing lots (n=5). The mice were injected subcutaneously in the right back with 0.2 ml MDA-MB-231 cells or MDA-MB-231 stem cells at concentrations of 10^6^, 10^5^, 10^4^ and 10^3^ cells/ml. The remaining 20 mice were divided into four experimental groups (n=5). The mice were injected subcutaneously in the right back with 0.2 ml MDA-MB-231 stem cells transduced with negative interference RNA (RNAi), Oct4 RNAi and Nanog RNAi constructs at concentrations of 10^6^ cells/ml. Mice were injected subcutaneously into the right back with 0.2 ml MDA-MB-231 stem cells without siRNA transfection, as a control group. After four weeks injection, all mice were sacrificed by cervical dislocation and tumor nodules were confirmed by necropsy. All experiments were approved by the Regional Ethical Committee for Animal Experimentation at Xiamen University.

### Statistical analyses

SPSS version 16.0 (SPSS, Inc., Chicago, IL, USA) was used to analyze the data. All data are expressed as the mean values or as the percentages of control values ± standard error of the mean depending on the experiments performed. Comparisons between two groups were calculated using Student’s t-test (two-tailed, independent) and comparisons among more than two samples were analyzed using one-way analysis of variance. P<0.05 was considered to indicate a statistically significant difference.

## Results

### Isolation and identification of CSCs from MDA-MB-231 breast cancer cell lines

#### Differential growth patterns of MDA-MB-231 breast CSCs

**Figure 1:**
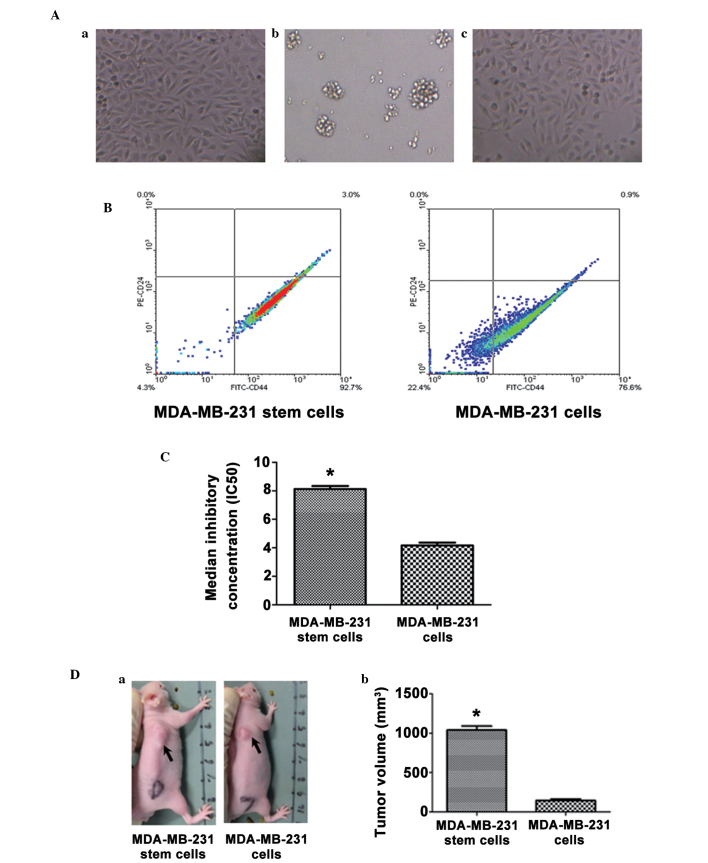
Isolation and identification of cancer stem cells from MDA-MB-231 breast cancer cell lines. (A) Culture of (Aa) MDA-MB-231 cells in medium with 10% fetal bovine serum (FBS), (Ab) MDA-MB-231 stem cells in serum-free medium and (Ac) MDA-MB-231 stem cells in medium with 10% FBS. (B) Fluorescence-activated cell sorter analysis of cluster of differentiation 44 (CD44)/CD24 expression in MDA-MB-231 breast cancer cell lines cultured in serum-free medium (MDA-MB-231 stem cells) and in medium with 10% FBS (MDA-MB-231 cells). The majority of MDA-MB-231 stem cells (97.2%) exhibited positive CD44 staining and negative CD24 staining, which was significantly higher than the percentage of MDA-MB-231 breast cancer cells (76.6%) with the corresponding staining pattern. The analysis was repeated three times. (C) Cell inhibition curves of the median inhibitory concentration (IC) of paclitaxel following the incubation of MDA-MB-231 stem cells and MDA-MB-231 cells with paclitaxel solution. The data are representative of three independent experiments (means ± standard error of the mean). ^*^P<0.05, as compared with the MDA-MB-231 cells. (D) Tumor xenografts in mice. (Da) The arrows indicate visible tumors induced by MDA-MB-231 stem cells and MDA-MB-231 cells, respectively. (Db) The volume difference of tumors induced by MDA-MB-231 stem cells and those induced by MDA-MB-231 cells. ^*^P<0.05 compared with the MDA-MB-231 cells (n=5).

Human MDA-MB-231 breast cancer cells were plated into 96-cell culture dishes in DMEM medium with 10% FBS and cultured adherently (Fig. 1Aa). After 48 h culture in serum-free medium supplemented with B27, EGF and bFGF, the cells that were adherent to the dish died and left behind spherical clusters formed in suspension, which were subsequently defined as MDA-MB-231 stem cells (Fig. 1Ab). After 12 h culture in differentiating medium with 10% FBS, the floating cells were able to re-adhere and differentiate (Fig. 1Ac).

#### Elevated percentage of the CD44^+^CD24-subpopulation in the mammosphere cell population

As breast cancer progenitor cells have been previously identified as CD44^+^CD24^−/low^ cells, the cellular expression of CD44 and CD24 was evaluated by flow cytometry. The majority of the MDA-MB-231 stem cells (97.2%) exhibited positive staining for CD44 and negative staining for CD24, which was a significantly higher percentage than that of the MDA-MB-231 cells (76.6%; Fig. 1B).

#### Isolated mammosphere cell resistance to paclitaxel inhibition

The stem cell phenotype of the isolated mammosphere cells was further verified by the high resistance of the cells to chemotherapy. The result revealed cell inhibition curve of MDA-MB-231 stem cells and MDA-MB-231 cells following exposure to paclitaxel solution. The cell inhibition rate was dose-dependent. In the MDA-MB-231 stem cells, 30 μg/ml paclitaxel was required to reach a 100% cell inhibition rate; however, the MDA-MB-231 cells only required 15 μg/ml paclitaxel to reach a 100% cell inhibition rate. In the MDA-MB-231 stem cells, the paclitaxel IC_50_ value was almost two-fold higher than that of the MDA-MB-231 cells (8.13±0.21 vs. 4.17±0.20 μg/ml; P<0.05; Fig. 1C).

#### High tumor-initiating capability of isolated mammosphere cells

To compare the tumorigenicity of the MDA-MB-231 stem cells and the MDA-MB-231 breast cancer cells, the two types of cell were injected subcutaneously into NOD/SCID mice. After four weeks, MDA-MB-231 cells gave rise to novel tumors when at least 0.2×10^6^ cells per animal were injected; however, at lower cell doses, no tumors developed. By contrast, the MDA-MB-231 stem cells formed tumors in five out of five, three out of five and one out of five animals when 0.2×10^6^, 0.2×10^5^ and 0.2×10^4^ cells/animal were injected, respectively (Table III). When equal quantities (0.2×10^6^ cells/animal) of cells were injected, the MDA-MB-231 stem cells formed significantly bigger tumors than the MDA-MB-231 cells (1,040.00±49.80 vs. 146.20±16.48 mm^3^; P<0.05 Fig. 1D).

### Higher expression levels of the Oct4 and Nanog transcriptional factors in the MDA-MB-231 stem cells, as compared with the MDA-MB-231 cells

**Figure 2:**
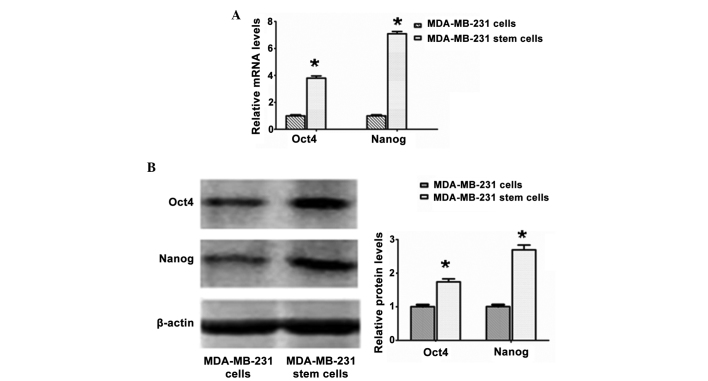
Expression levels of the octamer-binding protein 4 (Oct4) and Nanog transcriptional factors in MDA-MB-231 breast cancer stem cells. (A) Quantitative polymerase chain reaction reveals the relative expression levels of Oct4 and Nanog in MDA-MB-231 cells and MDA-MB-231 stem cells. (B) The protein expression levels of Oct4 and Nanog were determined by western blot analysis. Data are representative of three independent experiments (mean ± standard error of the mean). ^*^P<0.05 compared with the MDA-MB-231 cells.

The MDA-MB-231 stem cells exhibited significantly higher relative mRNA and protein expression levels of the Oct4 and Nanog putative stem cell markers than the MDA-MB-231 cells (P<0.05), which was further confirmed by real-time PCR (Fig. 2A) and western blot analysis (Fig. 2B).

### Reduced MDA-MB-231 stem cell drug resistance to paclitaxel following downregulation of Oct4 and Nanog

#### Expression levels of Oct4 and Nanog mRNA are reduced when either Oct4 or Nanog is knocked-down

**Figure 3:**
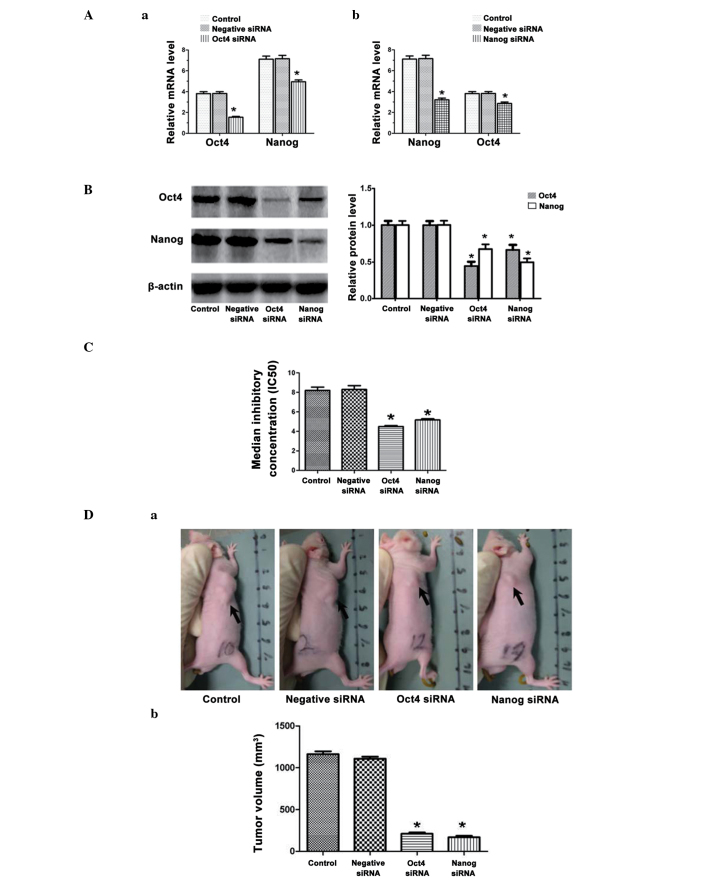
(A) Efficacy and the specificity of targeted small interfering (si)RNA silencing of (Aa) octamer-binding protein 4 (Oct4) and (Ab) Nanog gene expression analyzed by real-time polymerase chain reaction (PCR) analysis 48 h after siRNA transfection of MDA-MB-231 breast cancer stem cells. (B) The protein expression levels of Oct4 and Nanog following siRNA transfection of the MDA-MB-231 stem cells were determined by western blot analysis. (C) Paclitaxel cell inhibition curves following MDA-MB-231 stem cell transfection with negative siRNA, Oct4 siRNA or Nanog siRNA, and subsequent incubation with paclitaxel solution. The data are representative of three independent experiments (means ± standard error of the mean). ^*^P<0.05, as compared with the control and negative siRNA. (D) Reduced tumor-initiating capability of MDA-MB-231 stem cells following downregulation of Oct4 or Nanog in mice. (Da) The arrows indicate visible tumors induced by MDA-MB-231 stem cells transfected with negative siRNA, Oct4 siRNA or Nanog siRNA. (Db) The difference in volume of tumors induced by MDA-MB-231 stem cells transfected with different siRNAs. ^*^P<0.05 compared with the negative RNAi and control groups.

Oct4 and Nanog mRNA and protein downregulation following transfection of MDA-MB-231 stem cells with the respective RNAi molecules were analyzed by qPCR and western blot analysis, respectively. shRNA transduction of Oct4 constructs not only significantly reduced Oct4 mRNA but also significantly downregulated Nanog transcripts in the MDA-MB-231 stem cells (P<0.05; Fig. 3Aa). Similarly, the MDA-MB-231 stem cells transfected with the Nanog RNAi constructs exhibited significantly reduced the expression levels of Nanog mRNA and significantly downregulated Oct4 mRNA (P<0.05; Fig. 3Ab). The results were consistent with the data concerning the respective protein molecules in the western blot analysis (all P<0.05; Fig. 3B).

#### Reduced drug resistance and tumor-initiating capability of mammoshere cells following downregulation of Oct4 and Nanog

The MDA-MB-231 stem cells transfected with Oct4 or Nanog RNAi constructs became more sensitive to paclitaxel inhibition. The data revealed paclitaxel inhibition curves for MDA-MB-231 stem cells or MDA-MB-231 stem cells transduced with negative RNAi, Oct4 RNAi and Nanog RNAi constructs (Fig. 3C). In the MDA-MB-231 stem cells transfected with the Oct4 RNAi constructs, the IC_50_ values were almost two-fold lower than those of cells transfected with negative RNAi (4.49±0.10 vs. 8.30±0.39 μg/ml; P<0.05). The MDA-MB-231 stem cells transfected with Nanog RNAi constructs also exhibited reduced IC_50_ values, as compared with the cells transfected with negative RNAi (5.17±0.12 vs. 8.30±0.39 μg/ml; Fig. 3C). Therefore, the data demonstrated that downregulation of Oct4 or Nanog enhanced the sensitivity of human breast CSCs to drug chemotherapy. Furthermore, the tumorigenicity of the MDA-MB-231 stem cells transfected with Oct4 RNAi or Nanog RNAi was reduced. When injecting equal quantities of cells, the Oct4 RNAi and Nanog RNAi groups formed significantly smaller tumors than either the negative RNAi or control group (1,163.00±33.80 and 1,108.00±24.93 mm^3^ vs. 210.80±16.60 and 167.80±17.76 mm^3^; P<0.05; Fig. 3D).

## Discussion

Breast cancer is currently the most frequently occurring type of cancer and the primary cause of cancer-related mortality in females worldwide (10). With increasing advances in the investigation of CSCs, breast CSCs have been gradually determined to be capable of self-renewal and maintaining tumor growth and heterogeneity, as well as being rare, rendering these cells a promising foundation for stem cell-based therapeutics (25–28). Therefore, the identification of pure breast CSCs is key for the development of targeted antitumor therapies. In the present study, mammospheres were observed to form in serum-free medium in the presence of B27, EGF and bFGF, and with sustained long-term suspension culture. In medium with the addition of 10% FBS, mammosphere cells are able to differentiate into numerous cell types *in vitro*. The results from the present study support the CSC hypothesis that stem cells in culture are characterized by a self-renewing and proliferation ability upon appropriate stimulation, as well as by an undifferentiated status and the capacity to differentiate into heterogeneous mature cell types, results comparable with those observed by Li *et al* (27). Even through breast CSCs have been reported to be CD44^+^/CD24^−/low^ cells, it is not sufficient to define a stem cell solely on its surface markers (8,9,29). In the present study, mammosphere cells were demonstrated to predominantly consist of the CD44^+^CD24^−/low^ subpopulation. However, MDA-MB-231 breast cancer cells also had 76.6% CD44^+^CD24^−/low^ cells, indicating that the CD44^+^CD24^−/low^ subpopulation may encompass stem cells with self-renewal and other cell types without this property (29). Furthermore, the isolated mammosphere cells were also revealed to be more tumorigenic *in vivo* and more refractory to chemotherapy than the original MDA-MB-231 breast cancer cells, which was consistent with the results of previous studies (12,25,27). These data revealed that the isolated mammosphere cells were true breast CSCs, thus the cells were termed MDA-MB-231 stem cells.

In recent years, increasing evidence has emerged that CSCs exert an important role in drug resistance, tumor relapse and cancer metastasis in various types of cancer, including breast cancer (13,14). Major factors that affect drug sensitivity include drug-associated gene variation, the expression of the ATP binding cassette family of membrane transport proteins and the expression of antiapoptotic genes (30–33). Oct4 and Nanog are core transcriptional factors within the regulatory network required for the maintenance of self-renewal and pluripotency in embryonic stem cells, and any upregulation or downregulation induces divergent cell fates (22). Either Oct4 or Nanog depletion may result in the differentiation of normal human pluripotent stem cell cultures (34). Previous studies have observed that Oct4 and Nanog are overexpressed among numerous malignant solid tumor types that are immortal, undifferentiated and invasive (19,23). Knockdown of the two factors may inhibit tumor development and growth (20,22). Thus, Oct4 and Nanog may serve as a regulatory code for the response of breast CSCs to drug therapy. In concurrence with the results of previous studies (23,24), the MDA-MB-231 stem cells in the present study exhibited relatively high expression levels of the Oct4 and Nanog. Furthermore, the IC_50_ values were shown to be almost two-fold lower than those of the controls when the MDA-MB-231 stem cells were transfected with Oct4 RNAi constructs. MDA-MB-231 stem cells transfected with Nanog RNAi constructs also exhibited reduced IC_50_ values as compared with the controls, demonstrating that downregulation of Oct4 or Nanog enhanced the sensitivity of the human breast CSCs to drug chemotherapy. Furthermore, when injecting equal quantities of cells into mice, the MDA-MB-231 stem cells transfected with Oct4 RNAi or Nanog RNAi formed significantly (P<0.05) smaller tumors than the negative RNAi or control group cells, demonstrating that the downregulation of Oct4 or Nanog reduced the tumorigenicity in breast CSCs. Therefore, the present study indicated that Oct4 or Nanog-targeted therapy may be a promising means of overcoming resistance to chemotherapy and inhibiting tumor growth in breast cancer.

In conclusion, breast CSCs were isolated by suspension culture in serum-free medium and human breast CSCs were characterized with elevated percentages of the CD44^+^CD24^−/low^ subset, high tumorigenicity and resistance to chemotherapy, which encompassed stem cell-like properties. Furthermore, breast CSCs also expressed high levels of the Oct4 and Nanog transcriptional factors. To the best of our knowledge, the present study revealed for the first time the key role of Oct4 and Nanog in chemotherapeutic resistance and tumor growth in breast CSCs, which provides a possible novel insight into stem cell-based target therapies in breast cancer.

## Figures and Tables

**Table I:** Primer sequences used in the quantitative polymerase chain reaction experiments.

Gene	Primer sequence (5′-3′)	Amplicon size (bp)
Oct4	Forward: 5′-AGCAAAACCCGGAGGAGT-3′ Reverse: 5′-CCACATCGGCCTGTGTATATC-3′	114
Nanog	Forward: 5′-TGAACCTCAGCTACAAACAG-3′ Reverse: 5′-TGGTGGTAGGAAGAGTAAAG-3′	124
GAPDH	Forward: 5′-GAAGGTGAAGGTCGGAGTC-3′ Reverse: 5′-GAAGATGGTGATGGGATTTC-3′	226

Oct4, octamer-binding protein 4.

**Table II:** siRNA sequences used for silencing in the RNA interference experiments.

siRNA target gene	Primer sequence (5′-3′)	Molecular weight
Oct4	Forward 5′-GGAUUAAGUUCUUCAUUCATT-3′	21 bp/4943.32
	Reverse 5′-UGAAUGAAGAACUUAAUCCCA-3′	21 bp/5540.69
Nanog	Forward 5′-UGAUUGUUCCAGGAUUGGGTG-3′	21 bp/5257.49
	Reverse 5′-CACCCAATCCTGGAACAATCA-3′	21 bp/6407.11

siRNA, small interfering RNA; Oct4, octamer-binding protein 4.

**Table III:** Tumor-initiating capability of isolated MDA-MB-231 stem cells and MDA-MB-231 cells.

	Tumorigenicity	
	
Cells/ animal	MDA-MB-231 stem cells	MDA-MB-231 cells
0.2×10^6^	5/5	3/5
0.2×10^5^	3/5	0/5
0.2×10^4^	1/5	0/5
0.2×10^3^	0/5	0/5
